# Development of a fast and sensitive RT-qPCR assay based on SYBR® green for diagnostic and quantification of Avian Nephritis Virus (ANV) in chickens affected with enteric disease

**DOI:** 10.1186/s12917-024-03881-8

**Published:** 2024-01-30

**Authors:** Anthony Loor-Giler, Sara Castillo-Reyes, Silvana Santander-Parra, Manuel Caza, Nikolaos C. Kyriakidis, Antonio J. Piantino Ferreira, Luis Nuñez

**Affiliations:** 1 Facultad de Ingeniería y Ciencias Aplicadas, Carrera de Ingeniería en Biotecnología, Universidad de Las Américas (UDLA), Antigua Vía a Nayón S/N, Quito, EC 170124 Ecuador; 2 Facultad de Ciencias de La Salud, de Las Américas (UDLA), Carrera de Medicina Veterinaria, UniversidadAntigua Vía a Nayón S/N, Quito, EC 170124 Ecuador; 3 Facultad de Medicina, Cancer Research Group, Universidad de Las Américas (UDLA), Quito, 170504 Ecuador; 4 Department of Pathology, School of Veterinary Medicine, University of Sao Paulo, Av. Prof. Dr. Orlando M. Paiva, 87, Sao Paulo, SP 05508-270 Brazil; 5 One Health Research Group, Universidad de Las Américas, Quito, Ecuador

**Keywords:** RT-qPCR, Enteric viruses, ANV, SYBR Green, diagnosis

## Abstract

**Background:**

Enteric viruses are among the most prominent etiological agents of Runting-Stunting Syndrome (RSS). The Avian Nephritis Virus (ANV) is an astrovirus associated with enteric diseases in poultry, whose early diagnosis is essential for maintaining a good poultry breeding environment. ANV is an RNA virus that rapidly mutates, except for some conserved regions such as ORF1b. Therefore, the approach of a diagnostic method based on fast-RT-qPCR using SYBR® Green that focuses on the amplification of a fragment of ORF1b is presented as a feasible alternative for the diagnosis of this viral agent. In this study, the proposed assay showed a standard curve with an efficiency of 103.8% and a LoD and LoQ of 1 gene viral copies. The assay was specific to amplify the ORF 1b gene, and no amplification was shown from other viral genomes or in the negative controls. 200 enteric (feces) samples from chickens (broilers) and laying hens with signs of RSS from Ecuadorian poultry flocks were examined to validate the proposed method.

**Results:**

Using our method, 164 positive results were obtained out of the total number of samples run, while the presence of viral RNA was detected in samples collected from one day to 44 weeks old in both avian lines.

**Conclusions:**

Our study presents a novel, rapid, robust, and sensitive molecular assay capable of detecting and quantifying even low copy numbers of the ANV in commercial birds, therefore introducing a handy tool in the early diagnosis of ANV in enteric disease outbreaks in poultry.

## Background

Astroviruses are considered important human and animal pathogens as they cause gastroenteritis and other enteric diseases in infected individuals [1]. The Astroviridae family is divided into two genera: Mamastrovirus for mammals and Avastrovirus for birds [2]. Currently, two species of Avastroviruses infect chickens. They are associated with adverse effects on their growth and development and with the pathogenesis of enteritis and other lesions in the gastrointestinal and urogenital tract of young individuals [3]. The first is chicken astrovirus (CAstV), and the second is avian nephritis virus (ANV). Both viruses are implicated in disease pathogenesis in young and hatchery birds, being especially common in broilers [4].

The genus Avastrovirus is divided into three groups numbered 1 to 3, and ANV is located in the second group of Avastroviruses [4]. ANV was initially classified as a picornavirus, however, molecular characterization of the complete genome of the virus reclassified it within the astroviridae family (Imada 2000) [5]. ANV is a small, round, non-enveloped virus with a genome composed of single-stranded positive-sense RNA of approximately 6800 nucleotides in length [6]. The genome of this virus has three open reading frames (ORFs) located within untranslated regions and a 3' poly-A tail [7, 8]. The three coding regions are designated ORF1a, which generates small peptides that make up a viral protease series, ORF1b encoding two non-structural polyproteins (nsp1a and nsp1b), which are precursors of structural proteins necessary for genome replication, and ORF2 which encodes the structural polyprotein of the virus capsid [6]. Currently, three ANV variants have been identified by immunofluorescence and virus neutralization assays, namely ANV-1, 2, and 3. Amongst the different ANV variants, the most conserved region of the ANV genome is found within ORF1b [9]. On the other hand, since ORF2 is the most variable and discriminatory region among the virus variants, it has been widely used for virus genotyping. However, the different ANV serotypes have a considerable genetic variation, providing them with varying infection properties [10]. The high genetic diversity of RNA astroviruses is a byproduct of the accumulation of point mutations that have led to the emergence of different functional mechanisms, thus creating new variants [11].

ANV was first described in 1979 (Imada 1979) [12], where young chicks with severe interstitial nephritis and consequently high mortality were reported [13–15]. Since this report, the virus was widely described around the world as a causal agent of kidney problems [16–20]. ANV, in addition to being described in problems associated with kidney damage, where the affected chicks appeared dwarf, with ruffled feathers and a large amount of urates in the cloaca, the virus began to be detected in animals that, in addition to the signs mentioned, presented signs of enteric disease, mainly diarrhea, resulting in the virus being associated with RSS [21–28].

Enteric diseases have significant consequences in animal growth and are especially detected in the poultry industry. In poultry, runting-stunting syndrome (RSS) substantially impacts the gastrointestinal homeostasis of birds. Since many viruses are involved in its pathogenesis, it is also known as infectious stunting syndrome [26, 27].

This syndrome is characterized by dwarfism, poor organism development, diarrhea, poor digestion and malabsorption of nutrients, and high culling of birds at an early age [19].

RSS pathogenesis has been linked with infections by chicken astrovirus CAstV, fowl adenovirus (FAdV), chicken parvovirus (ChPV), infectious bronchitis virus (IBV), avian rotavirus group A (AvRT), avian reovirus (ARV) and ANV [19, 21, 22, 25]. ANV was detected in the feces of chicks affected with enteric disease, and with the classic clinical signs of animals affected with RSS [22, 23, 25, 28]. ANV as well as other viruses related to RSS are capable of reproducing the syndrome when inoculated alone or in combination with other viruses associated to RSS [19, 29–31]. ANV mainly affects young animals, being capable of producing enteric disease in the first 2 weeks of life, and maintaining viral dissemination for several weeks, even being found in adult animals, several weeks old [19].

A growing body of literature presents ANV as an emerging pathogen implicated in the occurrence of RSS and other enteric disorders, making the development of diagnostic methods for detecting and quantifying this pathogen of paramount importance [22, 32], where several molecular assays was development using the ORF1b gene as an affordable region to use as a target for detect ANV, due to this gene is shown as the most conserved region of Astrovirus genome [12, 13], and all genotypes of ANV showed this gene region with high similarity of nucleotides [4].

Herein, we developed and validated a rapid, sensitive, inexpensive diagnostic method for detecting and quantifying ANV based on RT-qPCR assays using SYBR® Green. Since molecular methods have shown high specificity and sensitivity for diagnosing viral diseases, they are efficient for analyzing and quantifying viruses causing diseases of current impact on animal and human health. This technique could be readily and massively used to help diagnose and treat early millions of infected chickens worldwide and thus significantly impact animal health.

## Results

### Primers

**Fig. 1:**
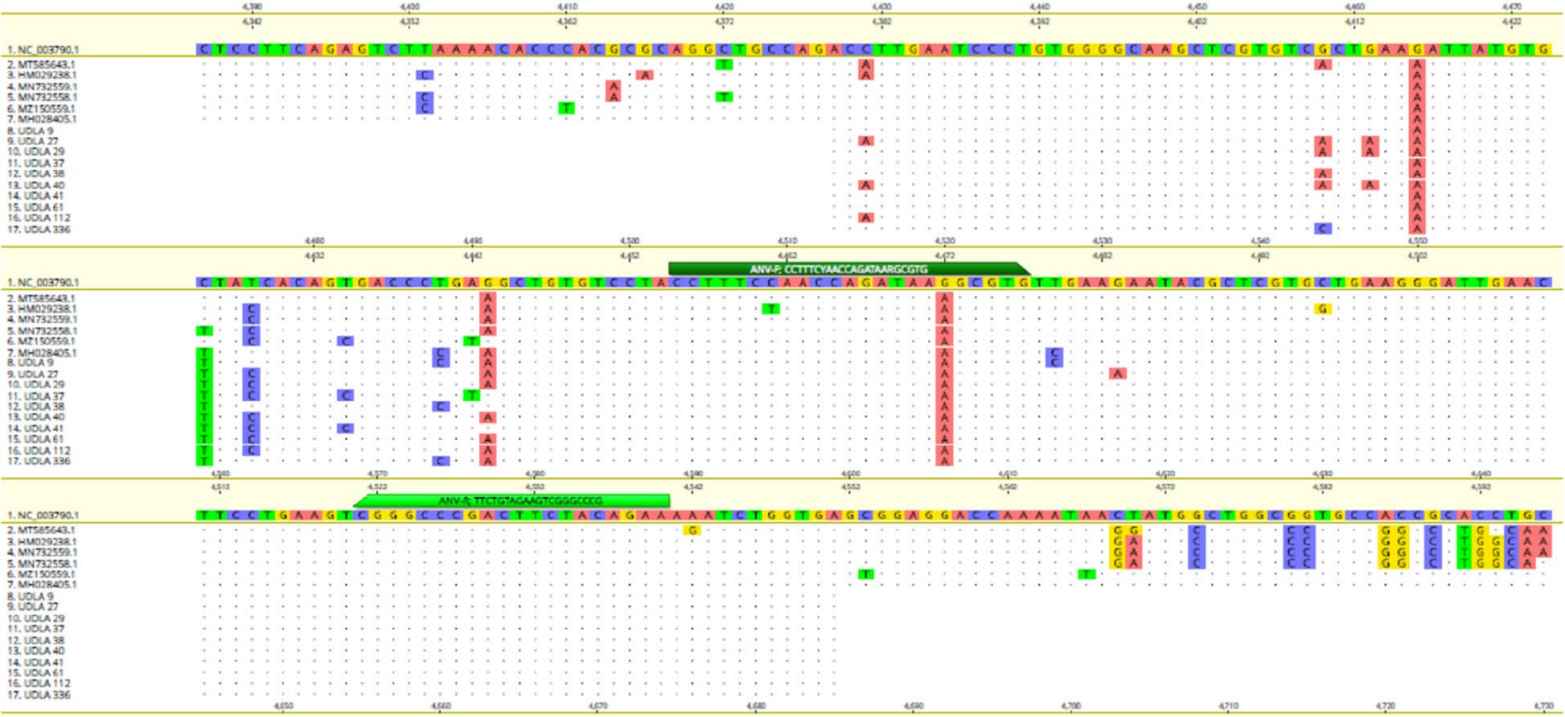
Alignment built with the sequences (NC_003790.1;MT585643.1; HM029238.1; MN732559.1; MN732558.1; MZ150559.1; MH028405.1) used for primer design and the sequences here obtained. The bars in green (different shades) on the reference sequence indicate the location of the primers

The primers could amplify an amplicon of 86 bp that flanked the last part of ANV's ORF 1b gene (Table 1). The primer ANV-F is located between nucleotide (NT) 4455 – 4477, and the primer ANV-R is located between NT 4521—4540 based on the reference sequence NC_003790.1 (Fig. 1).

**Table 1:** Primers used in this study

Primer	Gene	Assay	Sequences	Product	Reference
ANV-F	ORF1b	qPCR	5’-CCTTTCYAACCAGATAARGCGTG -3’	86 bp	This study
ANV-R			5’-TTCTGTAGAAGTCGGGCCCG -3’

### Determination of standard curve

**Fig. 2:**
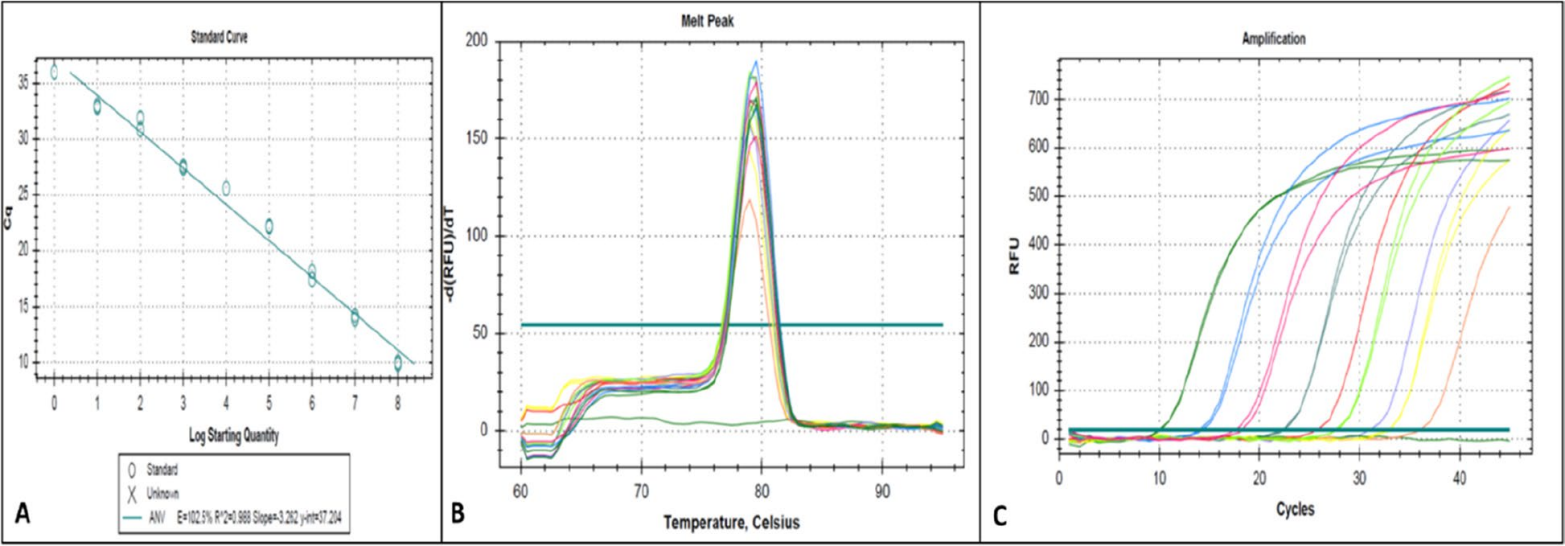
Real-time PCR with SYBR® FAST PCR double-strand DNA intercalating for specific detection and quantification of the conserved region ORF1b of ANV: **a** Efficiency curve, **b** Melting curve, and Amplification plot **c**

The ten dilutions used built a standard curve with an efficiency of 103.8%, a slope of -3.233, and a correlation coefficient of 0.988 (Fig. 2A). No primer dimers were observed in any run.

### Limit of detection and quantification

The standardized method detected up to 10^8^ copies of the DNA (Fig. 2B). The LoD and LoQ were one target gene copy (Fig. 2A). The melting curve showed a single peak without any alterations (Fig. 2C) and a melting temperature of 79,50 °C. Any amplification were obtained in the no template control reactions, and no primer dimers were present.

### RT-qPCR run time

The run time of the qPCR in fast conditions lasts approximately one hour, and with the melting curve analysis, it is about one and a half hours, reaching 2 h by adding the time that the RT test takes. The same assay with standard conditions extends to around two hours without the melting curve analysis and the time of RT.

### Specificity of RT-qPCR assay

Next, we run a specificity validation assay for the RT-qPCR method using samples where viral nucleus acid of several viruses such as ChPV, CAstV, IBV, AMPV, and FAdV were previously detected and sequenced. This assay clearly showed an exclusive amplification of the ORF1b fragment of the ANV positive control belonging to previously sample detected and sequenced of the virus, without any non-specific amplification of sequences of the other viruses tested.

### Repeatability of assay

Repeatability analysis performed with g-Block dilutions from 10^8^ to 10^4^ copies showed an inter-assay CV of 0.387 to 0.976% and an intra-assay CV of 0.085 to 0.430% (Table 2).

**Table 2:** Repeatability assays using g-Block dilutions from 10^8^ to 10^4^ copies of genetic material

Copy Number	Inter-Assay		Intra-Assay	
	**Cq Mean**	**Cq St Dev**	**Cq Mean**	**Cq St Dev**
10^8^	14,424	0,743	13.965	0,430
10^7^	19,661	0,765	16,932	0,303
10^6^	21,768	0,976	21,20	0,094
10^5^	25,024	0,767	24,21	0,107
10^4^	29,781	0,387	27,18	0,085

### Evaluation of RT-qPCR Assay for Detection of ANV

The fast-RT-qPCR-based detection method using SYBR Green for ANV diagnosis detected the presence of ANV viral copies in 164 of 200 samples from chickens with RSS (Table 3), leaving only 36 samples with undetected ANV. Our RT-qPCR analysis demonstrated that the highest average number of gene copies per microliter of RNA (GC/uL) was found in broilers up to 21 days old with 52,924 GC/uL and a maximum value quantified in the 21 days old broiler group of 561,785 GC/uL. The broiler group showed its lowest average viral copies in chickens up to 41 days old with 499 GC/uL and increased again in the group of chickens older than 41 days old with 3298 GC/uL. In the group of breeder hens, the highest average viral copies were found in hens older than 44 weeks with 666,310 GC/uL, the highest average reported in the whole study. The maximum value quantified in the group of breeder hens was found in those up to one week, with 5,538,297 GC/uL being the highest quantified of the whole study. The group of hens up to 30 weeks presented the lowest average quantified of the experiment with 77 GC/uL and the lowest number of positive samples (Table 3). Samples collected from the breeder group showed an increase in average viral load with increasing weeks from hens older than one week to older than 44 weeks. All the positive samples showed a melting temperature of 79.5 °C. No primer dimers were present in any run.

**Table 3:** Summary of ANV detection and quantification results in enteric samples from chickens with RSS

RT-qPCR Results for ANV detection and quantification						
**Birds**	**Age**		**Total samples**	**Positive samples**	**Average of GC/µL RNA**	**Maximum GC/µL RNA**
**Broilers**	**Days**	1–7	200	16/18	34,937	482,513
		> 7–21		11/17	52,924	561,785
		> 21–41		21/29	499	5329
		> 41		74/77	3298	95,241
**Breeders hens**	**Weeks**	1		16/21	371,997	5,538,297
		> 1–30		3/12	77	138
		> 30–44		10/11	16,193	128,545
		> 44		13/15	666,310	3,712,673

### Sequencing and phylogenetic analyses

**Fig. 3:**
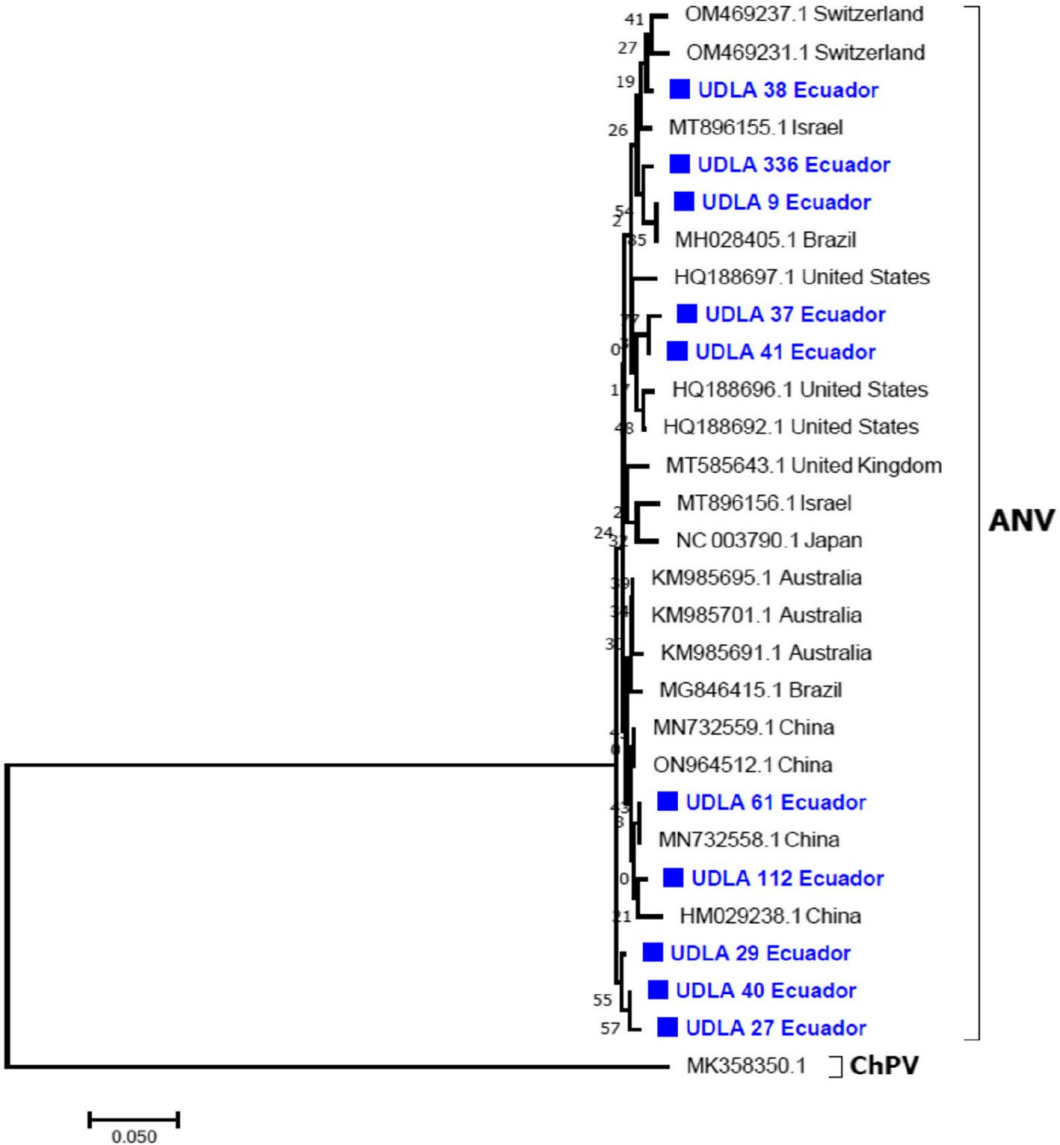
Phylogenetic relationships between the sequences of ANV obtained here and other sequences of ANV from Australia, Brazil, China, Israel, Japan, Switzerland, the United Kingdom, and the United States based on a part of ORF 1b gene NT sequences. Sequences were aligned using the CLUSTAL W method in ClustalX2 2.1. The phylogenetic tree was inferred using MEGA7 software on the alignments of the partial ORF 1b sequences of ANV using a Phylogeny reconstruction with a Neighbor-joining Statistical Method joined with a p-distance model and 1000 bootstraps of replication. The tree showed the phylogenetic relationships of the Ecuadorian ANV sequences with others present in GenBank. The numbers along the branches indicate the bootstrap value for every 1000 replicates. The scale bar represents the number of substitutions per site. The Chicken Parvovirus sequence (MK358350.1) was used as an outgroup. ■ = Sequences obtained in this study and written in blue

The present study showed ten arrangements with 173 bp of part of ORF 1b gene of ANV (Fig. 1); all sequences were submitted to the Genbank under the number access above described. The obtained series begins at NT 4379 and ends at NT 4551 based on the reference sequence NC_003790.1. The analyses with the BLAST tool demonstrated that the obtained arrangements have high similarity with other sequences of ANV. The phylogenetic analyses showed that the sequences here obtained were grouped with sequences from Switzerland and Israel (UDLA 38), Brazil (UDLA 336 and UDLA 9), United States (UDLA 37 and UDLA 41), China (UDLA 61 and UDLA 112), the sequences UDLA 29, 40 and 27 were grouped in a separate branch (Fig. 3).

**Fig. 4:**
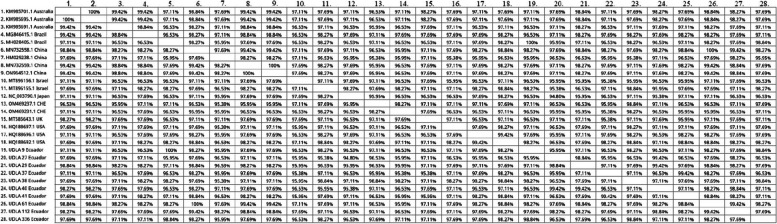
Comparison of the nucleotide identities of the sequences of Ecuadorian samples of ANV with other sequences of this virus. CHE = Switzerland; UK = United Kingdom; USA = United States

The sequences generated in the present study showed a 95,5 to 99,42% similarity of NT between them. The sequences obtained in the present study compared with other ANV sequences and showed a 96,53 – 98,84% NT similarity with different sequences of Australia, a 95,95 – 100% NT similarity with sequences from Brazil, a 95,38 – 100% NT similarity with sequences from China; a 95,38 – 98,84% NT similarity with sequences from Israel; a 94,8 – 97,11% NT similarity with sequences from Japan; a 95,38 – 98,84% NT similarity with sequences from Switzerland; a 95,38 – 97,69% NT similarity with sequences from United Kingdom; a 95,95 – 98,84% NT similarity with sequences from United States (Fig. 4).

### Statistical analysis

Chi-square statistical analysis showed a statistically significant difference in ANV presence between broiler and breeder hen samples (*p*-value = 0.001), so the rearing type could influence the infection's evolution. On the other hand, the analysis of a possible correlation between animal age and virus presence showed that age is statistically significantly associated with a higher occurrence of ANV only in the broiler group (*p*-value = 0.005).

## Discussion

Early diagnosis of the causative agents of enteric diseases such as ANV is pivotal to ensuring a disease-free poultry-rearing environment and minimizing animal and economic losses for producers. Thus, our diagnostic method based on a fast-RT-qPCR protocol using SYBR® Green for detecting and quantifying ANV emerges as a solution to this problem. Traditionally, the presence of specific viruses was detected mainly through electron microscopy. However, electron microscopy is a time-consuming and expensive method, making it inefficient for massive diagnosis [33]. Recent literature has proposed RT-qPCR as an alternative for diagnosing astroviruses such as ANV. However, hydrolysis probes' high cost and instability hinder their application and validation [34], and the use of NGS also demands high cost and in the country its use is limited. On the other hand, conventional PCR is a good tool for the detection of ANV [33], however, it requires more laboratory work and time for the release of the results, in addition to not allowing the quantification of viral particles.

The method proposed herein implies a lower price than the methods mentioned above by using SYBR® Green for the quantification of viral copies and a lower assay duration as it is a fast RT-qPCR. The standard curve obtained for ANV detection had an efficiency of 103.8%, demonstrating that it can be used to perform absolute quantification of viral copies in the sample run. Moreover, the melting curve analysis yielded the same temperature for all positive samples (79.5 °C), ensuring non-interference by non-specific products. When comparing the results obtained in this study with another assay where hydrolysis probes are used [34], it can be observed that the sensitivity of the assay proposed here is greater, being capable of detecting up to one copy of the virus, that is, if both assays show suitable efficiency curves. However, it is important to mention that although the efficiency of the curve is good and is within the optimal technical parameters for a qPCR assay, some contaminants present in the samples could interfere and alter the percentage efficiency of the standard curve, such as heparin, hemoglobin and undigested feed, contaminants that could be present in the intestinal contents of birds affected with digestive diseases, and that could alter the quality of the RNA and therefore alter the percentage efficiency of the standard curve.

Results obtained from RT-qPCR analysis of our test samples for AVN showed the highest average number of viral copies quantified in breeder hens older than 44 weeks with 666,310 GC/uL of RNA. This may be because RSS at such an advanced age may have induced a dysfunction of the organs of the gastrointestinal tract, leading to their conversion as reservoirs for enteric viruses such as ANV [35].

Both conditions could have been the cause of the initial development of the syndrome [18, 36]. Overall, the sample with the highest viral load was found in the group of breeder hens up to one week old with 5,538,297 GC/uL of RNA. This may be attributed to the fact that the virus infection already exists in the hen before hatching, since the vertical transmission of the virus from breeder hens to their eggs has previously been reported, causing an excessive proliferation of the virus in the hatched animal [37, 38]. On the other hand, we noted a progressive increment in the average viral load from the group of hens older than one week to the group of hens older than 44 weeks. One possible explanation for this observation is that a higher viral load can induce a more pronounced dysfunction of the organs of the gastrointestinal tract, which, in turn, as age advances, causes greater susceptibility to enteric diseases [39]. In the group of broiler chickens, the highest averages of quantified ANV particles were found in 2 groups: in the group up to 7 days old and in the group up to 21 days old, with 34,937 and 52,924 GC/uL of RNA, respectively, which was much lower than in the groups of broiler hens. A probable cause of this may be the fact that these birds, when produced for human consumption, receive well monitoring and better treatment, principally with antibiotics, probiotics, and prebiotics, which prevent secondary bacterial infections and improve the immune capacity of the individuals, therefore blocking viral propagation [40]. However, in our study, the most significant number of RSS-positive samples corresponded to broiler chickens. Of the total samples analyzed, 141 corresponded to broiler chickens, and the remaining 59 samples to breeder hens. This discrepancy shows a greater susceptibility to enteric diseases in broilers, possibly due to various broiler mechanisms since these have been implicated in the pathogenesis of conditions that affect poultry development and adequate growth [29, 41]. Additionally, it may also be attributed to the fact that the causal agent of RSS is another enteric virus, and the presence of ANV may be a byproduct of the development of the syndrome itself [42]. However, experimental infection of 1-day-old broiler chickens with ANV isolates led to enteric disease development characterized by diarrhea, apathy, ruffled feathers, cloacal pasting, dwarfism, and poor organ development [29]. ANV was initially associated with renal disease characterized by interstitial nephritis that caused high mortality, but, as earlier mentioned, ANV was detected and isolated in chickens with enteric disease [26]. RSS is related to several viruses (CAstV, IBV, FAdV, ChPV, ARV, ANV), detected alone or in many combination [5, 13, 16, 17]. Experimentally, was determined that any of these viruses alone or in combination are capable to reproduce this syndrome [12], so, the molecular detection of each one of these viruses could lead the farmers to take biosafety standards in order to avoid viral spread [15, 22], and also check the sanitary status of chicks in the first seven days of life, due to the vertical transmission that some of them present [17], in this context the RT-qPCR here propose shown as a very important tool for early diagnostic of RSS. Phylogenetic analysis showed the distribution of the various ANV sequences detected in this study as a function of the region used. These showed a broad distribution compared to sequences from several countries where ANV has been reported [43–46]. When analyzing the differences between the sequences obtained in this study and the sequences previously reported in the NCBI, it was identified that the NT sequences have remarkable similarity between them than the AA sequences; however, since they are not complete CDS, more information is needed in subsequent studies to be able to discriminate their behavior [20, 47, 48].

In the present study, viral RNA was detected in the fezzes of broiler chickens and breeder hens of different ages, in line with other studies where the virus was also detected in birds affected with enteric disease [22]. Overall, the method proposed in the present study highlights the possibility of using a fast-RT-qPCR assay based on SYBR® Green for detecting and quantifying ANV in poultry that could inform the development of appropriate interventions for early disease control.

## Conclusion

The present study shows the development of a fast RT-qPCR-based diagnostic method using SYBR® Green for detecting and quantifying ANV from low to high viral concentrations, with a high specificity for its target gene. Moreover, the assay demonstrated the presence of ANV in Ecuadorian chicken flocks of different age groups in both broiler and breeder hens. Thus, the assay presented here could be a high-throughput diagnostic molecular tool for detecting and quantifying ANV in poultry worldwide.

## Methods

### Sampling and RNA extraction

For the present study, we used 200 feces samples from dead chickens [broiler (141) and breeder hens (59)] with signs of enteric disease, mainly RSS, sent to UDLA research laboratories for necropsy. These samples were previously subjected to molecular screening (PCR and RT-PCR) for enteric viruses (ChPV, IBV, AvRT, ARV and FAdV), in, and some were found to be positive for ANV. The selected samples were rescreened for ANV by molecular analysis using the RT-qPCR assay presented herein, thus allowing for the RT-qPCR assay to be standardized and validated. Both sampling and all experimental procedures conducted for the present investigation were approved by the Committee for the Care and Use of Laboratory and Domestic Animal resources of the Agency of Regulation and Control of Phytosanitary and Animal Health of Ecuador (AGROCALIDAD), under the authorization serial number #INT/DA/019.

RNA was extracted from 100 mg of feces using TRIzol reagent (Invitrogen, Carlsbad, CA, USA) according to the manufacturer’s instructions.

### Primer design and standard DNA construction

For this study, we used two pairs of primers oriented to amplified a part of Orf 1b gene to detect the presence of ANV (Table 1). Primer design was carried out using the Geneious Prime 2022.1.1 software package with an alignment of the following complete ANV genome sequences found in NCBI until the present date: HM029238; MT585643; MN732558; MH028405; MZ150559; MN732559 and NC_003790 for newly reported genomes. Using these sequences to choose the primers ensures the method's detection capacity for the reported ANV variants.

To construct the standard curve, a g-Block (Integrated DNA Technologies – IDT, USA) was designed with the chosen sequence target for the primers. This g-block was dissolved according to the instructions of the distribution company and quantified in the Nanodrop 2000® (Thermo Fisher Scientific) spectrophotometer to determine the DNA concentration. The DNA Copy Number and Dilution Calculator web tool was used to calculate the quantity of recombinant DNA necessary to make the first dilution with a known amount of DNA copies. Tenfold dilutions from 10^8^ to 10^0^ copies were prepared to determine the sensitivity and amplification efficiency of the RT-qPCR assay.

### RT-qPCR assay

For ANV detection, a 2-step RT-qPCR was performed. For cDNA obtention, 8uL of RNA extracted from samples was subjected to reverse transcription using the SuperScript™ III Reverse Transcriptase (Invitrogen™ Van Allen Way, Carlsbad, California, USA) with 1uL of oligo (dT)20 (50 µM) and 1 µM of random hexamer primer according to the conditions and concentrations recommended by the manufacturers. The qPCR reaction was performed using a final volume of 10uL. Therefore, we used 5uL of PowerUp™ SYBR™ Green Master Mix 2x (Applied Biosystems by Thermo Fisher Scientific Vilnius, Lithuania), 0.7 uM of each primer (Table 1), 1uL of the cDNA and UltraPure™ DNase/RNase-Free Distilled Water dH2O (Invitrogen™ Van Allen Way, Carlsbad, California, USA) necessary to reach the final volume. The amplification protocol was set up in fast mode under the following conditions: Uracil-DNA glycosylase (UDG) activation step at 50 °C for 2 min, Dual-Lock DNA polymerase step at 95 °C for 2 min, and 40 cycles of double-strand denaturation at 95 °C for 3 s followed by 30 s at 60 °C for primer annealing and extension of the DNA template. The melting curve was generated by heating at 95 °C for 15 s, then progressive lowering of the temperature to 60 °C for 1 min and heating to 95 °C.

All 200 samples were subjected to the RT-qPCR protocol in the CFX96 Touch Real-Time PCR Detection System (Bio Rad Laboratories, Inc., Hercules, CA 94547, USA). All samples were run in duplicate, and absolute quantification was performed using the standard curve in each assay. Two non-template controls, two no reverse transcriptase controls and two negative extraction controls were added in each run.

### Limit of detection and quantification

The limits of detection and quantification were determined using the standard curve. The limit of detection (LoD) was defined as the lowest DNA concentration present in the ten-fold dilution series detected by the assay, and the lowest DNA concentration determined the limit of quantification (LoQ) that the assay could quantify and maintain within the linear portion of the standard curve.

### Repeatability of assay

For assessing the intra-assay and inter-assay repeatability and stability of the RT-qPCR assay, tenfold serial dilutions of the reference samples were prepared for RT-qPCR assay. The average value of Ct and the coefficient of variation (CV) were calculated according to the test results, and the stability of the assay was evaluated by CV.

Inter-assay repeatability: five tenfold serially diluted reference samples were amplified by RT-qPCR 5 times under the same reaction conditions. Intra-assay repeatability: five tenfold serially diluted reference samples were prepared, and five replicates were run for each dilution factor. The RT-qPCR assays were performed simultaneously.

### Specificity of the RT-qPCR assay

To determine the specificity of the ANV detection method developed, we run RT-qPCR assays using positive controls (samples where viral nucleus acids were detected) for the following viruses currently used in the laboratory of UDLA: ChPV, CAstV, IBV, avian metapneumovirus (aMPV) and FAdV. The positive controls were subjected to the same RT-qPCR assay developed in this study using the previously established conditions.

### cDNA Sequencing and phylogenetic analyses

To confirm that the current assay is binding and amplifying part of the ANV genome, ten samples where RNA of ANV was detected by the assay here development were randomly chosen and subjected to amplification of a portion of ORF 1B gene that includes the region where the primers were designed. So, for cDNA sequencing a part of the genome of ANV was amplified with the assay of RT-PCR described before by Tood et al., [49]. Then, one uG of RNA was subjected to RT reaction as described above. The cDNA obtained (2.5 uL) was submitted to a PCR containing the following: 0.5 µM of each primer, 2.5 µL of buffer 10 × , 0.5 µL of dNTPs 10 mM, 50 mM of MgCl2, 1.0 U of Platinum Taq DNA polymerase (Invitrogen by Thermo Fisher Scientific). The amplicons generated were purified using an ExoSAP-IT™ Express PCR product Cleanup (Applied Biosystems, Santa Clara, CA 95051, USA) as described by the manufacturer. Each purified product was sequenced in the forward and reverse sense using a BigDye® Terminator v3.1 Cycle Sequencing Kit (Thermo Fisher Scientific). In the present study, the assay described by Tood et al., 2011 flanked the last part of ORF 1b, and the complete ORF 2 gene was used uniquely for the part of the ORF 1b gene (173 bp). So, the obtained electropherograms were edited using the Geneious software package version 10.2.3 (Biomatters Ltd., Auckland, New Zealand) and analyzed using BLAST to determine if the sequences were similar to other ANV sequences deposited in GenBank.

Other sequences of ANV were downloaded from GenBank and were aligned with the obtained sequences using the ClustalX2 2.1 software package, and the phylogenetic analysis was carried out in MEGA 7 software [50]. The phylogenetic tree was built with a Neighbor-joining statistical method with a p-distance substitution model and phylogeny test bootstrap model with 1000 replicates.

### Statistical analysis

A descriptive analysis of the analyzed samples was carried out. A Chi-square test was carried out to find out if there are statistically significant differences in the presence of ANV between birds with different commercial purposes (broilers and breeders) in Jaimovi 2.3.24. Moreover, we carried out an analysis investigating whether the presence of the virus was associated with the age of the different groups of birds.

### GenBank accession numbers

The sequences obtained here of a part of the ORF 1b gene were submitted to the Genbank under the accession number: UDLA 9 (QR756280); UDLA 27 (QR756281); UDLA 29 (QR756282); UDLA 37 (QR756283); UDLA 38 (QR756284); UDLA 40 (QR756285); UDLA 41 (QR756286); UDLA 61 (QR756287); UDLA 112 (QR756288); UDLA 336 (QR756279).

## Data Availability

All data are included in this published article. The GenBank accession numbers of all sequences were listed in the material and methods section.

## Abbreviations

RSS: Runting-stunting syndrome
CAstV: Chicken astrovirus
FAdV: Fowl adenovirus
ChPV: Chicken parvovirus
IBV: Infectious bronchitis virus
AvRT: Avian rotavirus group A
ARV: Avian reovirus
ANV: Avian Nephritis Virus
GC: Gene Copies
NT: Nucleotides
AA: Amino acid
CDS: Coding sequence
